# New Perspectives on the Efficacy of Catgut Embedment in Acupoint Combined with Rehabilitation Training for Pediatric-Cerebral-Palsy Motor Function Disorders: A Systematic Review and Meta-Analysis of Randomized Controlled Trials

**DOI:** 10.3390/healthcare13111301

**Published:** 2025-05-30

**Authors:** Zhe-Hao Hu, Xin-Yue Zhang, Hong-Zhan Jiang, Xue-Jing Li, Yu-Fang Hao

**Affiliations:** 1 School of Nursing, Beijing University of Chinese Medicine, Beijing 100029, China; 20220521052@bucm.edu.cn (Z.-H.H.); 20220521048@bucm.edu.cn (X.-Y.Z.); 2 School of Traditional Chinese Medicine, Beijing University of Chinese Medicine, Beijing 100029, China; 20240941089@bucm.edu.cn

**Keywords:** cerebral palsy, motor function disorders, muscle spasticity, complementary therapies, catgut embedment in acupoint, meta-analysis

## Abstract

**Background**: Motor Function Disorders (MFDs) are common conditions in children with cerebral palsy and closely related to muscle spasticity. Catgut Embedment in Acupoint (CEA) has shown promise as an important adjunctive therapy but current evidence remains insufficient. The aim of this study was to evaluate the efficacy and safety of CEA in Pediatric-Cerebral-Palsy Motor Function Disorders (PCPMFDs). **Methods**: PubMed, Cochrane Library, Embase, Web of Science, four Chinese databases and two clinical trial registries were searched to include randomized controlled trials of patients with PCPMFDs treated with CEA combined with conventional rehabilitation. Meta-analysis was performed using Review Manager 5.4, Stata 18 and R Studio software 2025, and risk of bias was assessed for the included studies using the Cochrane Collaboration Network tool. **Results**: A total of 17 papers were included, including 1106 PCPMFDs patients with a wide range of conditions, age ≤ 9 years, and rehabilitation training mostly using Bobath/Vojta therapy. Meta-analysis showed that CEA was effective in improving MFDs with the Gross Motor Function Measure Scale (SMD, 0.90 [95% CI, 0.57 to 1.23], *p* < 0.0001) and the modified Ashworth Scale (MD, −0.40 [95% CI, −0.58 to −0.23], *p* < 0.0001). Preliminary results suggested that a treatment regimen, which consisted of three monthly sessions and lasted for one to two months, was most effective. **Conclusions**: CEA is an effective complementary treatment for patients with PCPMFDs with mild adverse effects. However, due to the relatively new perspective of this study, only a small number of researchers have focused on this area and conducted studies, resulting in fewer included studies meeting requirements, which is a direct result of the fact that this study, although informative, still requires a significant amount of research before clear evidence-based recommendations can be developed.

## 1. Introduction

Cerebral palsy (CP) is one of the most common causes of Motor Function Disorders (MFDs), caused by nonprogressive disturbances in the developing brain, and the relevant data show that the average frequency of CP is 2.08/1000 live births [1,2]. MFDs and impaired neuromusculoskeletal function are positively correlated, resulting in patients with CP that not only have their motor function severely affected but also suffer from muscle spasticity, which in turn severely reduces patients’ quality of life [3,4]. Therefore, the effective treatment and care of Pediatric-Cerebral-Palsy Motor Function Disorders (PCPMFDs) has become one of the hotspots of modern medical research.

At present, standardized treatment protocols for PCPMFDs have not been established. During the treatment, electrical stimulation and botulinum toxin are feasible treatment options [5]. In the treatment of muscle spasticity, baclofen is often used [6,7]. Moreover, in severe cases, neurosurgery is also included in the treatment program [7]. There is growing evidence that early rehabilitation training interventions and brain stimulation can be effective in curing neurological disorders [8,9]. However, current medications and surgeries have significant limitations and adverse effects, ranging from extensive muscle weakness and the inherent risks of surgery to prolonged recovery [10,11]. There is still a need for more effective and less invasive therapies, which makes complementary alternative medicine a potential area for further exploration.

**Figure 1:**
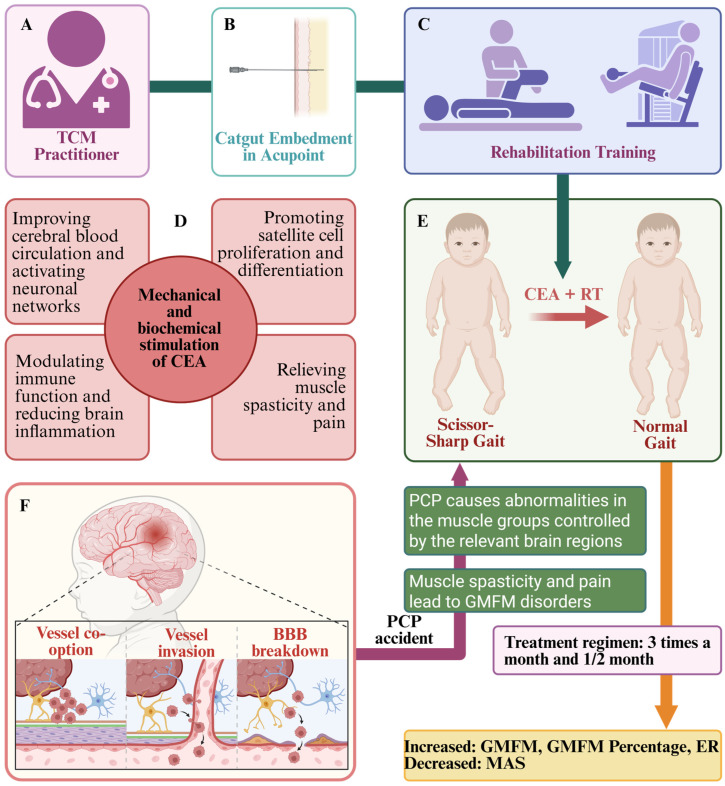
Graphical abstract. (**A**) Traditional Chinese medicine practitioners; (**B**) pictures of Catgut Embedment in Acupoint treatment; (**C**) rehabilitation training at different stages; (**D**) possible mechanisms of embedment in acupoint treatment to improve motor function disorders; (**E**) possible gross motor function disorders in Pediatric-Cerebral-Palsy patients; (**F**) possible brain damage in Pediatric-Cerebral-Palsy patients. Note: TCM, Traditional Chinese medicine; CEA, Catgut Embedment in Acupoint treatment; RT, rehabilitation training; PCP, pediatric cerebral palsy; BBB, blood-brain barrier; GMFM, Gross Motor Function Measurement Scale; GMFM Percentage, Gross Motor Function Measurement Percentage Scale; ER, effective rate; MAS, modified Ashworth scale.

Traditional Chinese medicine (TCM), especially acupuncture, has often been utilized in the treatment regimen on the basis of rehabilitation training (RT) and has shown good efficacy and high safety [12,13]. Catgut Embedment in Acupoint (CEA) is an important component of acupuncture therapy, which mainly involves the insertion of hydrolysable catgut into specific acupoints in order to produce continuous acupoint stimulation (Figure 1A,B). Unlike traditional acupuncture, CEA is characterized by relatively strong and long-lasting stimulation. According to TCM theory, these characteristics make it well suited for the treatment of brain disorders [14]. An increasing number of clinical studies have shown that the addition of CEA to treatment has better antispastic effects [15,16,17,18,19,20,21,22,23,24,25,26,27,28,29,30,31]. Zhang J [20] and Jin BX [32] suggested that CEA combined with other treatments is more effective in PCPMFDs, but due to clinical ethical issues, the relevant conclusions do not highlight the therapeutic mechanism, and there is still a lack of high-quality evidence-based studies and basic experiments. However, the evidence base for this observation remains unclear. In addition, its possible mechanism of improved CEA is also unclear.

## 2. Aim

This systematic review and meta-analysis aims to comprehensively evaluate the efficacy and safety of CEA as an important complementary therapy for PCPMFDs and synthesize all the available evidence to support its clinical application. Meanwhile, this study aims to explore the possible mechanisms of CEA to improve MFDs.

## 3. Methods

### 3.1. Protocol and Registration

This study followed the recommendations of the Cochrane Handbook for Systematic Reviews of Interventions [33] and the Preferred Reporting Items for Systematic Reviews and Meta-Analyses (PRISMA) statement [34]. The protocol had been previously registered at PROSPERO (ID: CRD420251022039).

### 3.2. Inclusion and Exclusion Criteria

The Participants, Interventions, Comparisons, Outcomes, and Study Designs (PICOS) framework was used to develop the inclusion criteria:

P (Participants): participants ≤ 12 years with a diagnosis of PCPMFDs based on clear diagnostic criteria or references were included in the study. There were no restrictions on gender, disease duration, or disease severity.

I (Interventions): the treatment program should be primarily centered around CEA and may be combined with other therapies (e.g., acupuncture). The original literature must provide a clear description of the CEA procedure, including details such as sterilization, frequency, duration, and post-treatment protocol.

C (Controls): the control group should be given the same treatment as the intervention group except for CEA.

O (Outcomes): the primary outcome was MFDs, assessed by one of the most commonly used clinical scales: the Gross Motor Function Measurement (GMFM) Scale, and from which the GMFM Percentage was extracted as data for subgroup analysis. Secondary outcomes included the effective rate (ER), the modified Ashworth scale (MAS) and adverse events (AEs).

S (Study Designs): only randomized controlled trials (RCTs) were included.

Exclusion criteria: (1) studies that lacked clear diagnostic criteria or caused MFDs by factors other than CP; (2) studies without primary outcome indicators; (3) investigations with incomplete outcome data; and (4) duplicate publications.

### 3.3. Search Strategies

Computerized searches were performed on PubMed, the Cochrane Library, Embase, Web of Science, Chinese Biomedical Literature Database (SinoMed), China National Knowledge Infrastructure (CNKI), Chinese Scientific Journal Database (VIP) and *Wanfang* Database. Meanwhile, manual searches of the Chinese Clinical Trials Registry and the US Health Ongoing Trials Registry (https://clinicaltrials.gov/) were conducted to identify potentially eligible trials. The searches were conducted from the establishment date to March 18, 2025 in each database. The search terms were mainly “Catgut Embedment in Acupoint”, “Cerebral Palsy”, and “Motor”. Two reviewers (ZH Hu and XY Zhang) independently screened the article titles and abstracts using EndNote 21. After abstract screening, the full text was downloaded for in-depth reading. If there was any disagreement between the two reviewers, an initial discussion was held. If the issue remained unresolved, the corresponding author of the relevant article would be consulted for clarification.

### 3.4. Data Extraction and Quality Assessment

Information extraction was performed by two evaluators (ZH Hu and XY Zhang) using a standardized data table. In case of disagreement, a third reviewer (XJ Li) will be asked to evaluate. Corresponding authors were contacted by e-mail if the necessary information was incomplete or unclear. The revised Cochrane Risk of Bias tool for RCTs, RoB 2.0 [35] was used to assess the risk of bias in the included studies. The tool assesses risk of bias in five domains: (a) bias in the randomization process; (b) bias in deviations from intended interventions; (c) bias in missing outcome data; (d) bias in outcome measurement; and (e) bias in the selection of the reported results. For each domain and overall judgment, each RCT was assigned one of three ratings: “low risk”, “some concerns” or “high risk”. Two assessors independently used this method to determine the risk of bias for each trial included [33]. Any disagreements were resolved by consensus.

### 3.5. Data Analysis

Review Manager 5.4, Stata 18 and R Studio software 2025 were used for data analysis. For primary and secondary outcomes excluding AEs, results were expressed as relative risk (RR) with its 95% confidence interval (CI). Effect estimates were calculated based on post-intervention values. Statistical analysis was performed according to the statistical guidelines specified in the latest Cochrane Handbook for Systematic Reviews of Interventions [33]. Meta-analysis was performed when trials showed good homogeneity in terms of participants, interventions, controls, outcomes and study designs. Meta-analysis was performed using a fixed-effects model when I^2^ ≤ 50% and *p* ≥ 0.1; otherwise, a random-effects model was used. If there was significant heterogeneity among studies (I^2^ > 50%), the source of heterogeneity was analyzed; if heterogeneity among studies was not significant (I^2^ < 50%), the optimal treatment strategy was explored. Subgroup analyses were performed using different types of controls.

## 4. Results

### 4.1. Study Selection

**Figure 2:**
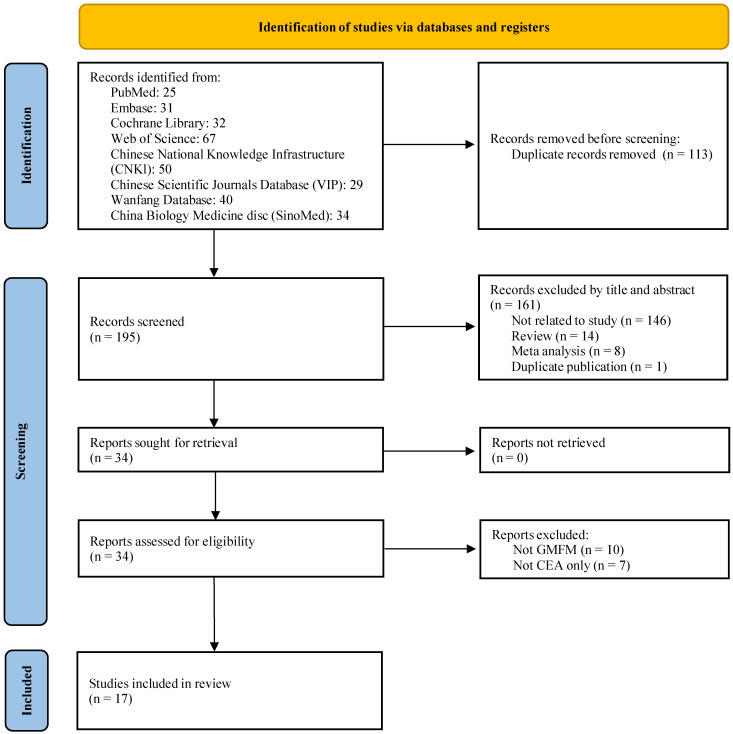
Flow diagram of the literature screening.

A total of 308 documents were retrieved. Subsequently, by filtering the titles and abstracts, only 34 articles remained. These 34 literatures were read in full text. After the final exclusion of 17 literatures, 17 literatures were included [15,16,17,18,19,20,21,22,23,24,25,26,27,28,29,30,31]. The screening process is shown in Figure 2.

### 4.2. Study Characteristics

Seventeen randomized controlled trials with a total of 1106 participants (438 in both treatment and control groups) were conducted, of which four trials [15,16,17,28] compared GVM + RT with acupuncture + RT, and two trials [20,27] compared CEA + acupuncture + RT with acupuncture + RT, and the remaining eleven trials directly compared CEA + RT with RT alone [18,19,21,22,23,24,25,26,29,30,31]. The duration of treatment in these trials was 1~3 months. All participants were children under 9 years of age. All trials were conducted in China and published in Chinese. Detailed characteristics of the included studies are shown in Table 1.

### 4.3. Risk of Bias of Included Studies

**Figure 3:**
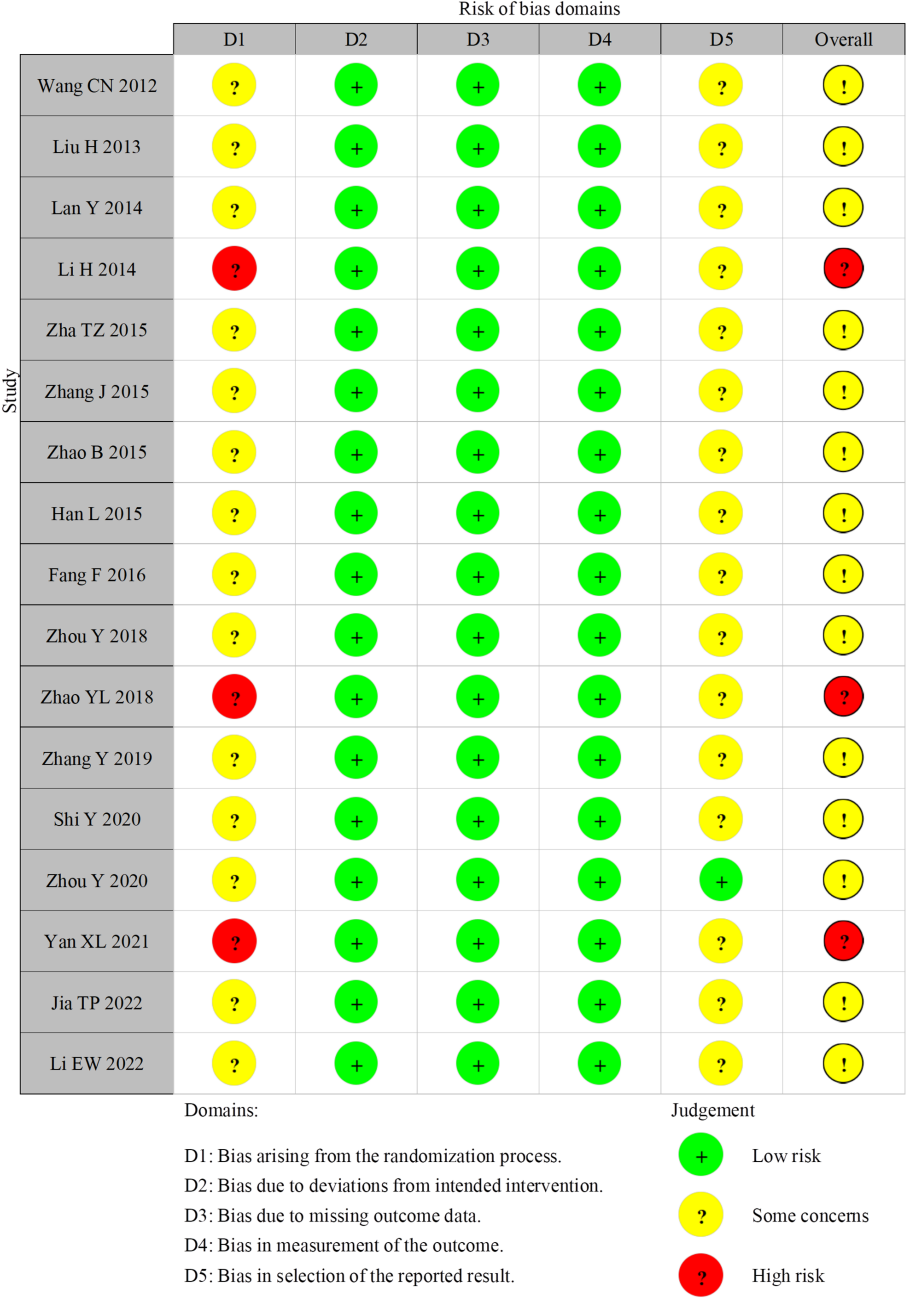
Summary of risk of bias judgments for each study [15,16,17,18,19,20,21,22,23,24,25,26,27,28,29,30,31].

A total of 17 RCTs were evaluated using the RoB 2.0 tool. Among them, three studies were identified as “high risk” [18,25,29], and the rest were identified as “some concerns” [15,16,17,19,20,21,22,23,24,26,27,28,30,31] (Figure 3). Overall, most studies were found to be moderate. Due to insufficient study details, 14 studies remained unclear about the risk of bias associated with allocation concealment because it was not clear whether the allocation order was concealed or not [15,16,17,19,20,21,22,23,24,26,27,28,30,31]. Three studies had a high risk of bias with respect to allocation due to the use of the order of hospitalization number for grouping [18,25,29]. Although no study [15,16,17,18,19,20,21,22,23,24,25,26,27,28,29,30,31] stated whether participants were aware of their group assignments during the trial, no study proposed additional interventions that deviated from the trial design. In total, 17 studies were free of shedding, suggesting a low risk of attrition bias [15,16,17,18,19,20,21,22,23,24,25,26,27,28,29,30,31]. Blinding poses significant challenges. Given the nature of CEA, blinding is often impractical and RoB 2.0 results showed unclear or poor-quality descriptions of blinding by participants and personnel. In addition, no RCTs noted that assessors blinded participants to the intervention [15,16,17,18,19,20,21,22,23,24,25,26,27,28,29,30,31]. Furthermore, only one study found supplemental data [28] and was low risk in terms of selective debriefing. The remaining studies lacked supplemental data, making it uncertain whether an analysis plan was developed prior to the start of each RCT [15,16,17,18,19,20,21,22,23,24,25,26,27,29,30,31]. The lower risk of bias of included studies, the more feasible in outcomes [33].

### 4.4. Primary Outcomes

The GMFM score ranges from 0 to 3 and a total of five functional areas were measured. The higher the score, the better the motor function [36,37]. However, the scoring criteria and final scores were not handled the same between studies, with some studies having data for only some of the functional areas, others using raw data, and most using percentage. To ensure analytical rigor and consistency, GMFM and GMFM Percentage were, respectively, used as the assessment of motor function.

#### 4.4.1. Effect on the GMFM

**Figure 4:**
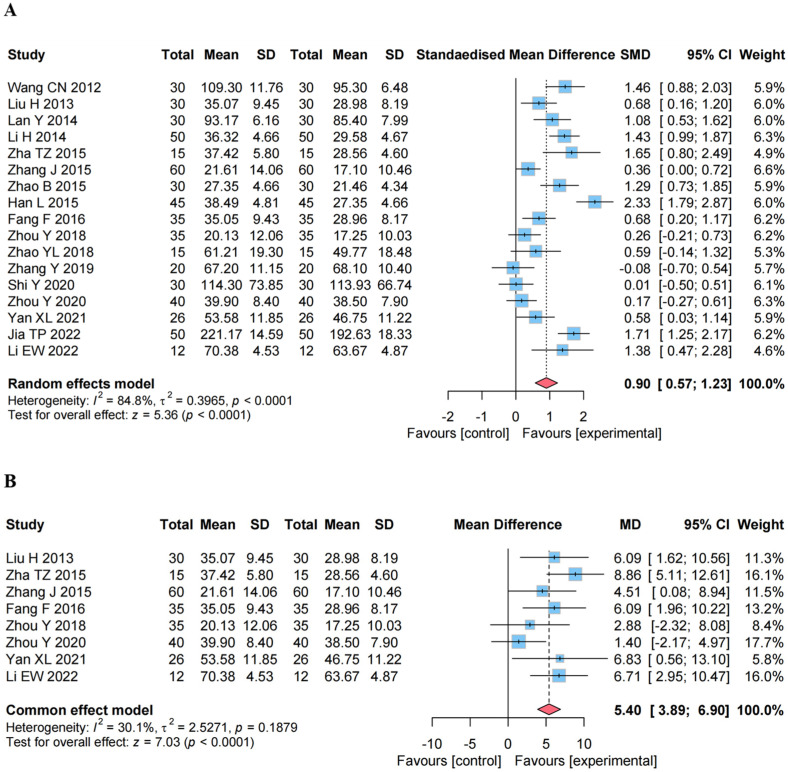
Forest plots of effects analysis of the primary outcomes. (**A**) Gross Motor Function Measure (GMFM) score [15,16,17,18,19,20,21,22,23,24,25,26,27,28,29,30,31]; (**B**) GMFM Percentage score [16,19,20,23,24,28,29,31].

As shown in Figure 4A, 17 studies using GMFM to examine motor function [15,16,17,18,19,20,21,22,23,24,25,26,27,28,29,30,31], with a total of 1106 participants, were analyzed using a random-effects model. The results of the meta-analysis showed that CEA combined with RT was more effective in improving GMFM scores (SMD, 0.90 [95% CI, 0.57 to 1.23], *p* < 0.0001), compared to the control group (RT alone). On average, the results obtained by treating the patients via CEA + RT is 0.90 units higher than in the control group (RT alone). The results also show that there is a 95% confidence that the true mean difference in the population lies between 0.57 and 1.23. Since the interval does not include 0, the result is statistically significant at the 5% level. According to the study of Fabio Alexander Storm, it can be concluded that the minimum clinically important difference of GMFM is between 0.1 and 3.0 units [38]. The results of the meta-analysis are within the interval and are clinically significant.

Sensitivity analysis regrouped the data by excluding one included study at a time. Seventeen studies were finally included. As shown in Supplementary Figure S3A, the combined results of the remaining 16 studies remained statistically significant after excluding any single study. The initial pooled results were minimally affected by the exclusion of individual studies. The sensitivity analysis is somewhat suggestive of robust and stable overall results.

#### 4.4.2. Effect on the GMFM Percentage

As shown in Figure 4B, the GMFM Percentage analysis included eight studies involving a total of 506 participants [16,19,20,23,24,28,29,31]. A fixed-effects model was used for the analysis. The results showed that the combination of CEA and RT was effective in improving patients’ motor function (I^2^ = 30.1%) by increasing GMFM Percentage (MD, 5.40 [95% CI, 3.89 to 6.90], *p* < 0.0001) compared to RT alone.

##### Sensitivity Analysis

As shown in Supplementary Figure S3B, the sensitivity analysis of GMFM Percentage indicated that the results appeared to be robust.

##### Subgroup Analysis

Given the small heterogeneity (I^2^ = 30.1%), we conducted more in-depth subgroup analysis based on several potential factors that may influence the outcome of real-world TCM treatment with a view to finding the most appropriate treatment regimen. These factors included treatment frequency and treatment duration.

As for treatment frequency, subgroup analysis showed that the GMFM Percentage score was significantly higher in the test group than in the control group (MD, 5.40 [95% CI, 3.89 to 6.90], *p* < 0.00001). Three treatments per month had the greatest effect size (MD, 7.19 [95% CI, 4.83 to 9.55], *p* < 0.00001).

In terms of treatment duration, the results showed that GMFM Percentage scores were significantly higher in the test group than in the control group with the assistance of CEA (MD, 5.40 [95% CI, 3.89 to 6.90], *p* < 0.00001). Notably, the effect size was greatest when the treatment duration was one to two months (MD, 4.26 [95% CI, 2.44 to 6.09], *p* < 0.00001). The results of the subgroup analysis are shown in Supplementary Figures S1 and S2.

### 4.5. Secondary Outcomes

#### 4.5.1. Effect on the ER

**Figure 5:**
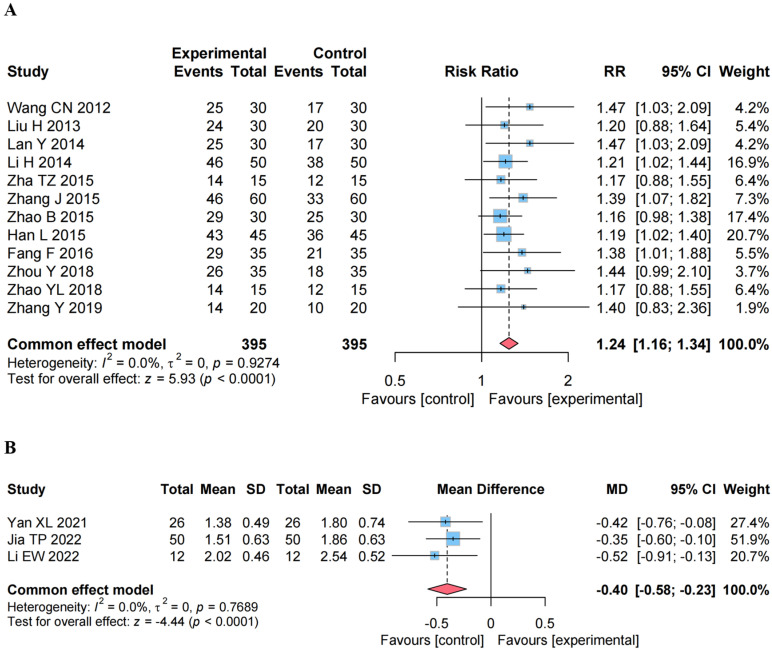
Forest plot of effects analysis of the secondary outcomes. (**A**) The effective rate (ER) [15,16,17,18,19,20,21,22,23,24,25,26]; (**B**) the modified Ashworth scale (MAS) score [29,30,31].

As shown in Figure 5A, 12 studies [15,16,17,18,19,20,21,22,23,24,25,26] calculated the effective rate (ER) of Motor Function Disorders in 506 patients with CP using a fixed-effects model. The results showed that CEA combined with RT significantly increased the ER compared with the control group (RR, 1.24 [95% CI, 1.16 to 1.34], *p* < 0.0001).

#### 4.5.2. Effect on the MAS

As shown in Figure 5B, three studies [29,30,31] used MAS to assess the degree of spasticity in patients with CP. The higher the MAS score, the more severe the muscle spasticity, using a fixed-effects model. The results of the meta-analysis showed that, compared with the control group, CEA combined with RT significantly decreased the MAS score (MD, −0.40 [95% CI, −0.58 to −0.23], *p* < 0.0001).

#### 4.5.3. Effect on the AEs

Only one study [20] reported adverse events associated with CEA. Two patients in the observation group of this study developed hard nodules at the site of the embedding catgut without redness, swelling or fever manifestations, which did not take any measures and subsided on their own in about 1 month.

#### 4.5.4. Sensitivity Analysis on Secondary Outcomes

As shown in Supplementary Figure S4, the sensitivity analysis of the ER, MAS scores indicates that the results appear to be robust.

### 4.6. Publication Bias

**Figure 6:**
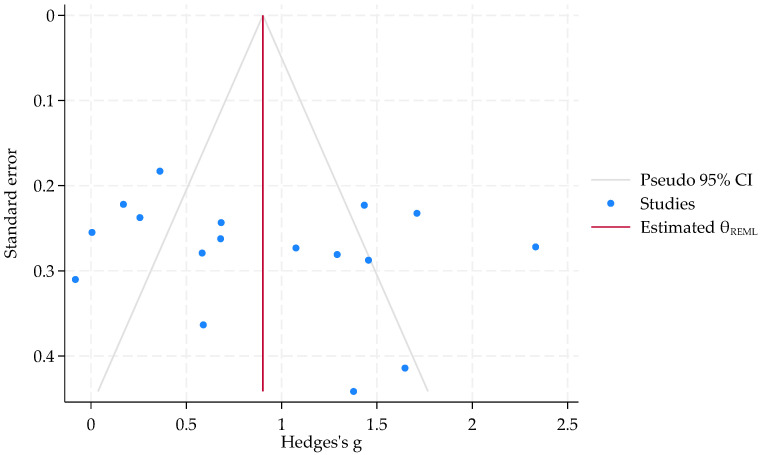
The funnel plot of publication bias of GMFM score.

Among the included studies, the GMFM score, which is the primary outcome indicator, was reported the most frequently—17 times [15,16,17,18,19,20,21,22,23,24,25,26,27,28,29,30,31]. Therefore, we assessed the publication bias of this indicator. No evidence of publication bias was found by Egger’s test (*p* = 0.3150 > 0.05) and Begg’s test (*p* = 0.3870 > 0.05) (Figure 6). More details can be found in the Supplementary Materials.

### 4.7. Evidence Evaluation

The certainty of the evidence was critically assessed. The results showed that the quality of the evidence for “ER” was moderate due to the risk of bias of the studies involved, and the evidence for the other three outcomes was of low quality. Specifically, “GMFM” was given this rating due to risk of bias and inconsistency between studies. The “GMFM Percentage” and “MAS” values were considered low-quality evidence due to the risk of bias and imprecision in data. A comprehensive summary of the results of the GRADE classification is summarized in Table 2.

## 5. Discussion

### 5.1. Summary of the Findings

To the best of our knowledge, this is the first study to conduct a rigorous systematic review and meta-analysis of 17 RCTs containing 1106 cases of PCPMFDs patients. The results suggest that CEA, as a low heterogeneity complementary therapy, shows great potential in improving PCPMFDs as measured by one of the most commonly used clinical scale (GMFM). In addition, it has been shown to be effective in reducing muscle spasticity (lowering MAS scores) in patients with CP. Only one study has reported an adverse event associated with CEA: sclerosis. This symptom resolved on its own without special care, suggesting that CEA has a high safety profile.

The results preliminarily suggest that CEA at three times per month and one-to-two-month sessions more significantly improve GMFM scores, due to the fact that CEA is characterized by relatively intense and prolonged stimulation, and that intensifying treatment frequency and treatment duration are more likely to induce tolerance to the acupoints, thus reducing the outcome [39,40]. Furthermore, this finding is consistent with most clinical situations, further validating its practical relevance in real-world applications. However, these results should be interpreted with caution given the relatively low quality of the overall evidence.

### 5.2. Perceptions of PCPMFDs

According to TCM, cerebral palsy belongs to the “five delays”, “five weaknesses” and “five stiffnesses” [41]. CEA can improve local microcirculation and regulate the muscle, bone and fascia system in the acupoint area, and reach the tissues and organs of the whole body through blood circulation, thus relieving local muscle spasticity and improving muscle strength [32].

In light of modern medical research, although the pathogenesis of PCPMFDs is not fully understood, recent studies have proposed the following potential mechanisms. (a) Immune function disorders: Overexpression of circulating cytokines in the brain (e.g., IL-1β, IL-6, TNF and CXCL8, etc.), along with the blockage of signaling pathways, such as ak-STAT and mTOR, which can regulate the amount of cytokines and circulating blood flow in the brain, ultimately affecting the development of the nervous system [42]. (b) Brain inflammation: Both astrocyte proliferation and myelin sheath damage can lead to neural hyperexcitability, ultimately causing brain inflammation [43]. (c) Muscle spasticity and pain: DNA methylation differences lead to damage to satellite cell differentiation that occurs early in life and inhibition of skeletal muscle growth and development, leading to increased deformity and pain as skeletal muscle grows [44]. (d) Receptor damage: Damage to proprioceptive receptors leads to impaired message transmission, which in turn affects the relevant muscle groups and ultimately MFDs such as balance and walking [45] (Figure 1E,F).

### 5.3. Possible Mechanisms of CEA to Improve MFDs

CEA is a hybrid therapy that combines mechanical and biochemical stimulation to produce therapeutic effects through the continuous stimulation of acupoints with hydrolysable catgut. Although the mechanisms by which CEA directly improve MFDs are not fully understood, relevant studies have shown that CEA can produce both systemic and local effects [14]. First, CEA uses absorbable catgut that act as a foreign protein and together with effective acupoint stimulation can create a synergistic effect that modulates immune function better than conventional acupuncture, as well as promoting blood circulation and collagen fiber formation [46,47] (Figure 1D). In addition, effective stimulation can repair the damage of brain white matter fiber bundles and promote the proliferation and differentiation of satellite cells, which in turn relieves the pain caused by muscle spasticity, and ultimately promotes the recovery of Gross Motor Function (GMF) [48,49]. Furthermore, it has been demonstrated that complementary alternative medicine (e.g., CEA, acupressure, moxibustion, etc.) triggers existing or new neuronal networks, as well as synaptic efficiencies or reactivation through highly increased sensory injury perception combined with proprioception [50].

### 5.4. Clinical Relevance

Compared with the same type of studies (e.g., Liu S [51] and Cheng YY [52]), the findings converge and corroborate with the results of this study. Not only that, the intervention in this paper was more focused, only including CEA and the sensitivity analysis was added while the heterogeneity was relatively low, together with the evaluation of heterogeneity using RoB 2, which increased the scientific validity and replicability of the present study. Ultimately, it was demonstrated that CEA + RT effectively improved patients’ motor function and was superior to RT alone. The above theoretical mechanisms belong to the summary of indirect evidence and there is little high-quality basic research on CEA for PCPMFDs. This paper provides directions for subsequent related studies.

### 5.5. Limitations and Implications for Future Research

Although CEA shows some promise in the treatment of PCPMFDs, there are still several aspects of the current study that may be improved. (1) There is no standardization of GMFM scores between studies, which can lead to high heterogeneity. These differences greatly increase the complexity of making direct comparisons of study results. (2) The sample sizes of the available studies appear to be somewhat limited. This may affect the comprehensiveness of representing different patient populations. (3) As the current studies were all conducted in China, the studies have a distinct geographical character. (4) The lack of long-term follow-up studies makes it difficult to determine the long-term efficacy of CEA.

To address these limitations, the following recommendations are proposed. Firstly, future studies should focus on organizing collaborative multicenter studies with long-term follow-up. Through this process, standardized CEA treatment protocols can be established together, with carefully defined important factors and scoring criteria that may affect clinical outcomes. Such standardization is essential to ensure comparability of findings across studies. In addition, musculoskeletal ultrasound can detect changes in muscle properties after CEA treatment [53], while functional magnetic resonance imaging (fMRI) provides a powerful tool for assessing the effects of CEA on neuromodulation in the brain [54]. The application of these advanced techniques may provide strong evidence for the role of CEA in improving PCPMFDs, which in turn may attract more patients from more countries willing to receive this TCM therapy.

Notably, a flexible and individualized rehabilitation program is highly recommended when integrating CEA (Figure 1C). Early rehabilitation is crucial, as infants reach a plateau in upper extremity function at baseline by 9–10 months [55]. With age, resistance training (to improve GMF) can be gradually introduced, such as sling exercise training, aquatic therapy and treadmill interventions [56,57,58,59]. During the chronic rehabilitation phase, the focus of rehabilitation should gradually shift from passive to active movement strategies. Range of motion training (aimed at maintaining and improving joint mobility) [60] and hybrid training (focused on increasing muscle strength) [59] are particularly beneficial. These research efforts are expected to facilitate the implementation of more effective and evidence-based CEA in PCP rehabilitation. Incorporating CEA into clinical practice allows practitioners to be more proactive in the rehabilitation of patients with PCPMFDs. This intervention promotes functional recovery, improves patients’ quality of life, and enhances overall clinical care. As such, it sets a higher standard for holistic, patient-centered TCM practice.

## 6. Conclusions

It is recommended that CEA be included in the comprehensive treatment of PCPMFDs. However, due to the relatively high risk of bias and distinct geographical character in the current study, the efficacy of this complementary therapy still needs to be verified more thoroughly. It is expected that relevant basic research will verify its role in improving motor function from a more scientific perspective.

## Figures and Tables

**Table 1:** The characteristics of included studies.

Study ID	Year	Sample Size (Male/Female)		Age of Patients		Outcomes	Treatment Frequency	Treatment Duration	Treatment Points
		E	C	E	C				
**CEA + RT vs. Acupuncture + RT**									
Wang CN	2012 [15]	30 (17/13)	30 (16/14)	2~6 (3.6 ± 0.5) years	2~6 (3.6 ± 0.5) years	(1)(3)	Once a week	3 months	Body Acupoints
Liu H	2013 [16]	30 (26/4)	30 (24/6)	6~24 (16.4 ± 2.5) months	6~30 (17.3 ± 2.7) months	(1)(2)(3)	Three times a month	3 months	Body/Scalp Acupoints
Lan Y	2014 [17]	30	30	(3.55 ± 0.4) years	(3.49 ± 0.5) years	(1)(3)	Once a week	3 months	Body Acupoints
Zhou Y	2020 [28]	40 (28/12)	40 (24/16)	6~33 (19.4 ± 6.6) months	8~35 (20.5 ± 7.4) months	(1)	Once a month	3 months	Body Acupoints
**CEA + Acupuncture + RT vs. Acupuncture + RT**									
Zhang J	2015 [20]	60 (40/20)	60 (36/24)	6~20 (14 ± 6) months	7~22 (14 ± 7) months	(1)(2)(3)(5)	Twice a month	3 months	Body Acupoints
Shi Y	2020 [27]	30	30	1~6 years	1~6 years	(1)	Three times a month	3 months	Body/Scalp Acupoints
**CEA + RT vs. RT**									
Li H	2014 [18]	50 (29/21)	50 (32/18)	12~40 (26.6 ± 8.2) months	12~48 (28.9 ± 6.8) months	(1)(3)	Once a week	3 months	Body Acupoints
Zha TZ	2015 [19]	15 (8/7)	15 (9/6)	(13 ± 2.3) months	(12 ± 2.1) months	(1)(2)(3)	Three times a month	1 month	Body Acupoints
Zhao B	2015 [21]	30 (19/11)	30 (12/18)	6~34 (13.6 ± 7.2) months	6~36 (15.9 ± 8.4) months	(1)(3)	Twice a month	3 months	Body/Scalp Acupoints
Han L	2015 [22]	45 (29/16)	45 (32/13)	6~34 (13.6 ± 7.2) months	6~36 (15.9 ± 8.4) months	(1)(3)	Twice a month	3 months	Body/Scalp Acupoints
Fang F	2016 [23]	35 (21/14)	35 (20/15)	6~30 (14.3 ± 5.2) months	7~30 (14.4 ± 5.5) months	(1)(2)(3)	Three times a month	3 months	Body/Scalp Acupoints
Zhou Y	2018 [24]	35 (20/15)	35 (18/17)	(47.3 ± 7.9) months	(48.2 ± 8.4) months	(1)(2)(3)	Twice a month	3 months	Body Acupoints
Zhao YL	2018 [25]	15 (11/4)	15 (10/5)	67~106 (81.3 ± 11.9) months	64~103 (80.2 ± l1.4) months	(1)(2)(3)	Once a week	2 months	Body/Scalp Acupoints
Zhang Y	2019 [26]	20 (10/10)	20 (9/11)	1~5 (3 ± 1) years	1~4 (3 ± 1) years	(1)(3)	Three times a month	3 months	Body/Scalp Acupoints
Yan XL	2021 [29]	26 (16/10)	26 (18/8)	1.42~4.33 (2.17 ± 0.67) years	1.25~4.42 (2.38 ± 0.87) years	(1)(2)(4)	Once a week	3 months	Body Acupoints
Jia TP	2022 [30]	50 (29/21)	50 (27/23)	1~7 (2.94 ± 0.75) years	1~6 (3.09 ± 0.52) years	(1)(4)	Once a week	3 months	Body/Scalp Acupoints
Li EW	2022 [31]	12 (8/4)	12 (6/6)	(4.2 ± 1.2) years	(4.0 ± 1.3) years	(1)(2)(4)	Twice a month	2 months	Body Acupoints

Note: E, experimental group; C, control group; CEA, Catgut Embedment in Acupoint; RT, Rehabilitation Treatment; (1) Gross Motor Function Measure (GMFM) Scale; (2) GMFM Percentage; (3) effective rate (ER); (4) modified Ashworth scale (MAS); (5) adverse events (AEs).

**Table 2:** GRADE classification for evidence evaluation.

Outcomes	No. of Studies	Certainty Assessment						No. of Patients		Effect		Certainty	Importance
		Study Design	Risk of Bias	Inconsistency	Indirectness	Imprecision	Other Considerations	E	C	Relative (95% CI)	Absolute (95% CI)		
GMFM	17	RCTs	serious ^a^	serious ^b^	not serious	not serious	none	553	553	-	SMD 0.90 higher (0.57 higher to 1.23 higher)	⊕⊕◯◯ Low ^a,b^	CRITICAL
GMFM Percentage	8	RCTs	serious ^a^	not serious	not serious	serious ^c^	none	253	253	-	MD 5.41 higher (3.52 higher to 7.29 higher)	⊕⊕◯◯ Low ^a,c^	CRITICAL
ER	3	RCTs	serious ^a^	not serious	not serious	not serious	none	335/395 (84.8%)	259/395 (65.6%)	RR 1.24 (1.16 to 1.34)	157 more per 1000 (from 105 more to 223 more)	⊕⊕⊕◯ Moderate ^a^	-
MAS	12	RCTs	serious ^a^	not serious	not serious	serious ^c^	none	88	88	-	MD 0.40 lower (0.58 lower to 0.23 lower)	⊕⊕◯◯ Low ^a,c^	-

Note: E, experimental group; C, control group; CI: confidence interval; MD: mean difference; RR: risk ratio; SMD: standardized mean difference; MAS, modified Ashworth scale; ER, effective rate; GMFM, Gross Motor Function Measurement Scale; GMFM Percentage, Gross Motor Function Measurement Percentage Scale; ⊕, the more “⊕”, the higher quality of evidence; ◯, the more “◯”, the lower quality of evidence; CRITICAL, Primary Outcomes; RCTs, randomized controlled trials; ^a^ lack of blinding; ^b^ I^2^ value of the combined results was large and high heterogeneity; ^c^ small sample size and the confidence intervals were wide.

## Data Availability

The data underpinning this study are available in the article and in its Supplementary Materials.

## Supplementary Materials

The following supporting information can be downloaded at: https://www.mdpi.com/article/10.3390/healthcare13111301/s1, Figure S1. The forest plot of subgroup analysis of treatment frequency; Figure S2. The forest plot of subgroup analysis of treatment duration; Figure S3. Forest plots of sensitivity analysis of the primary outcomes. (A) Gross Motor Function Measure (GMFM) score; (B) GMFM Percentage score; Figure S4. Forest plot of sensitivity analysis of the secondary outcomes. (A) The effective rate (ER); (B) the modified Ashworth scale (MAS) score; publication bias; search strategies.
