# Supplementary material for: New Perspectives on the Efficacy of Catgut Embedment in Acupoint Combined with Rehabilitation Training for Pediatric-Cerebral-Palsy Motor Function Disorders: A Systematic Review and Meta-Analysis of Randomized Controlled Trials
Source: Healthcare (Basel). 2025 May 30;13(11):1301. doi: 10.3390/healthcare13111301 (PMC12154081; doi:10.3390/healthcare13111301)
Supplement: Supplementary file 1 [file healthcare-13-01301-s001.zip › healthcare-3599541-Supplementary Materials.pdf]

New Perspectives on the Efficacy of Catgut Embedment in Acupoint Combined with Rehabilitation Training for Pediatric-Cerebral-Palsy Motor Function Disorders: A Systematic Review and Meta-Analysis of Randomized Controlled Trials

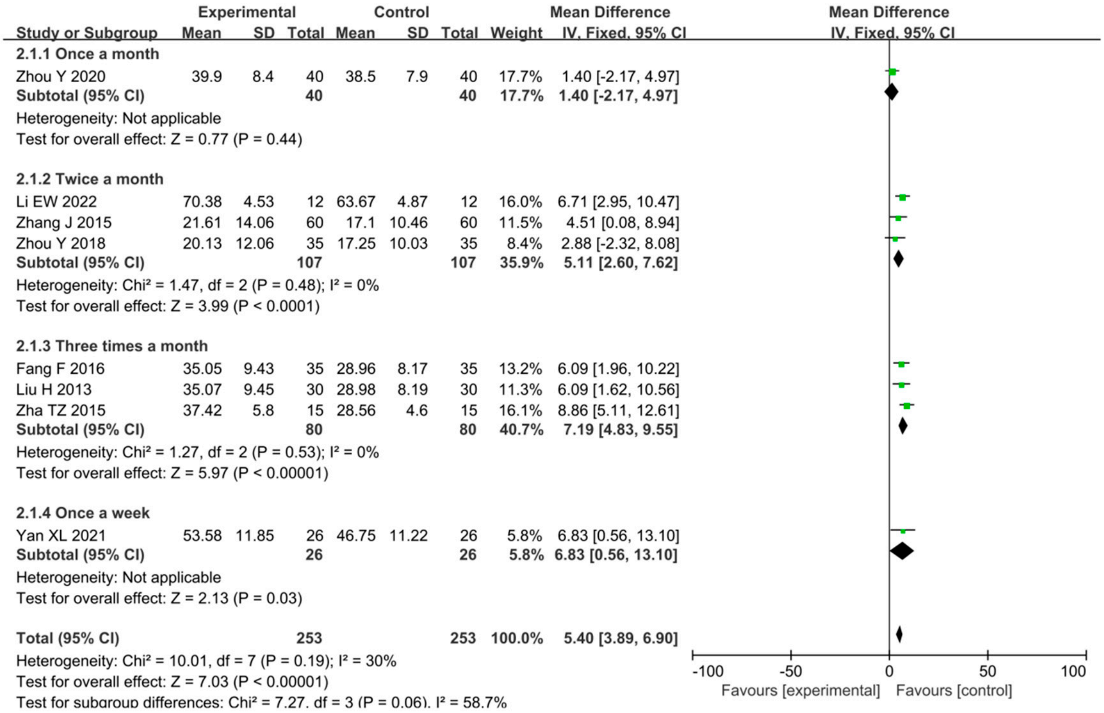

Figure S1. The forest plot of subgroup analysis of treatment frequency.

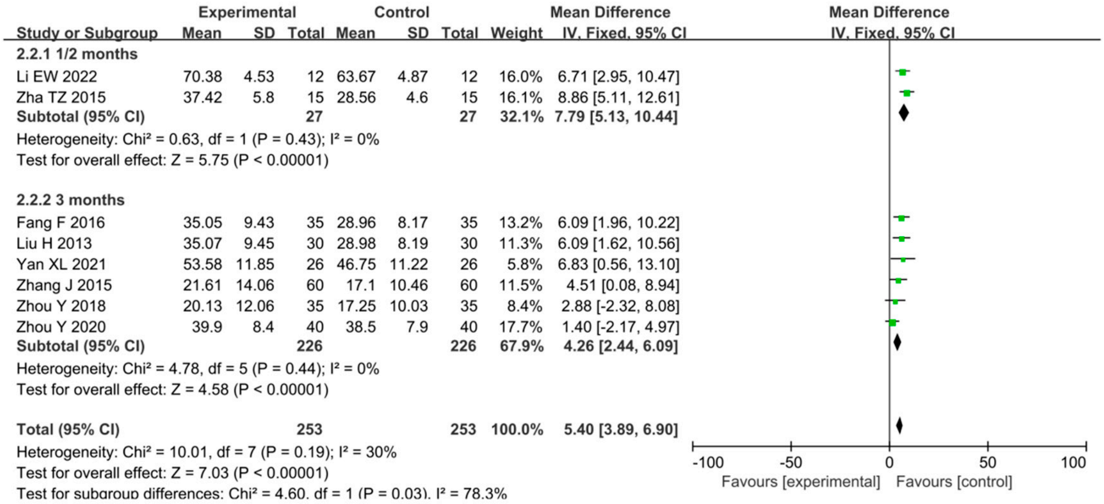

Figure S2. The forest plot of subgroup analysis of treatment duration.

A

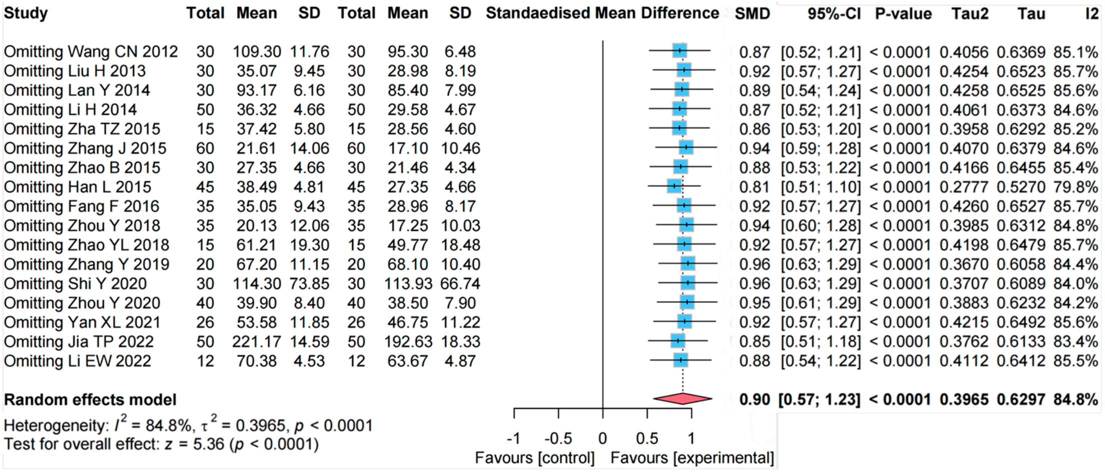

B

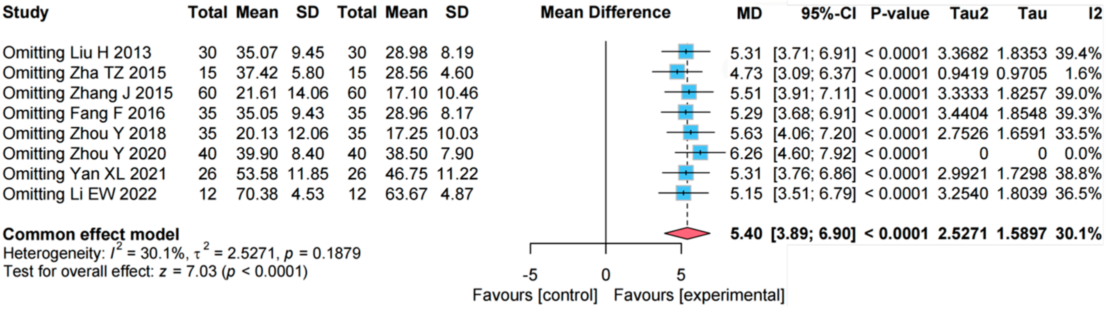

Figure S3. Forest plots of sensitivity analysis of the primary outcomes. (A) Gross Motor Function Measure (GMFM) score; (B) GMFM Percentage score.

A

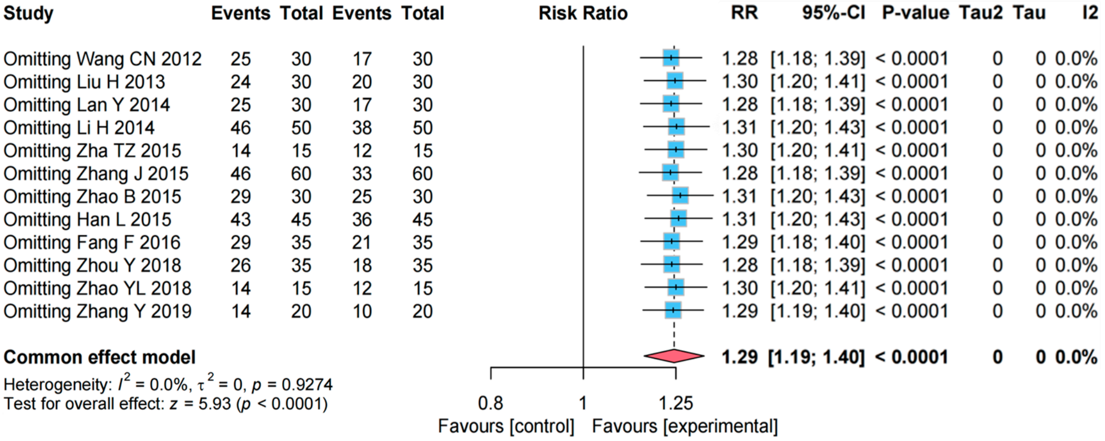

B

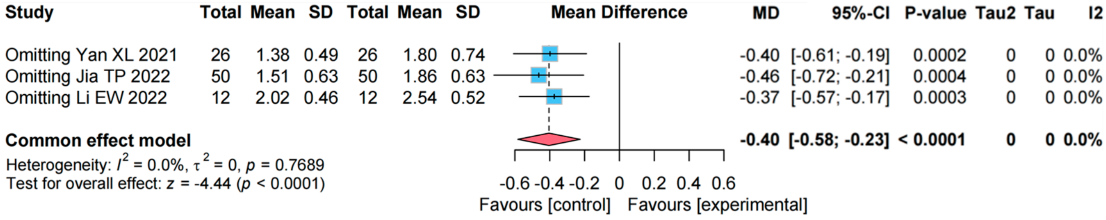

Figure S4. Forest plot of sensitivity analysis of the secondary outcomes. (A) The effective rate (ER); (B) The modified Ashworth scale (MAS) score.

Publication Bias

meta bias, egger

Effect-size label: Hedges's g
Effect size: _meta_es
Std. err.: _meta_se

Regression-based Egger test for small-study effects
Random-effects model
Method: REML

H0: beta1 = 0; no small-study effects
beta1 = 2.68
SE of beta1 = 2.665
z = 1.00
Prob > |z| = 0.3150

meta bias, begg

Effect-size label: Hedges's g
Effect size: _meta_es
Std. err.: _meta_se

Begg's test for small-study effects

Kendall's score = 22.00
SE of score = 24.276
z = 0.87
Prob > |z| = 0.3870

Search Strategies

S1

PubMed: 25

Filters: Randomized Controlled Trial

((("Cerebral Palsy"[Mesh]) OR (CP (Cerebral Palsy))) OR (Little Disease)) OR (Diplegia, Spastic)) OR (Little's Disease)) OR (Spastic Diplegia)) OR (Diplegias, Spastic)) OR (Spastic Diplegias)) OR (Cerebral Palsy, Atonic)) OR (Atonic Cerebral Palsy)) OR (Cerebral Palsy, Hypotonic)) OR (Hypotonic Cerebral Palsies)) OR (Hypotonic Cerebral Palsy)) OR (Cerebral Palsy, Athetoid)) OR (Athetoid Cerebral Palsy)) OR (Cerebral Palsies, Athetoid)) OR (Cerebral Palsy, Dyskinetic)) OR (Cerebral Palsies, Dyskinetic)) OR (Dyskinetic Cerebral Palsy)) OR (Monoplegic Cerebral Palsy)) OR (Cerebral Palsies, Monoplegic)) OR (Cerebral Palsy, Monoplegic)) OR (Monoplegic Cerebral Palsies)) OR (Cerebral Palsy, Quadriplegic, Infantile)) OR (Infantile Cerebral Palsy, Quadriplegic)) OR (Quadriplegic Infantile Cerebral Palsy)) OR (Cerebral Palsy, Congenital)) OR (Congenital Cerebral Palsy)) OR (Cerebral Palsy, Diplegic, Infantile)) OR (Diplegic Infantile Cerebral Palsy)) OR (Infantile Cerebral Palsy, Diplegic)) OR (Cerebral Palsy, Dystonic-Rigid)) OR (Cerebral Palsies, Dystonic-Rigid)) OR (Cerebral Palsy, Dystonic Rigid)) OR (Dystonic-Rigid Cerebral Palsies)) OR (Dystonic-Rigid Cerebral Palsy)) OR (Cerebral Palsy, Mixed)) OR (Mixed Cerebral Palsies)) OR (Mixed Cerebral Palsy)) OR (Cerebral Palsy, Rolandic Type)) OR (Rolandic Type Cerebral Palsy)) OR (Cerebral Palsy, Spastic)) OR (Spastic Cerebral Palsies)) OR (Spastic Cerebral Palsy)) OR (Cerebral Palsy, Monoplegic, Infantile)) OR (Infantile Cerebral Palsy, Monoplegic)) OR (Monoplegic Infantile Cerebral Palsy)))) AND (Embedding)

S2

Web of science: 67

Entitlements:

- WOS.IC: 1993 to 2025
- WOS.CCR: 1985 to 2025
- WOS.SCI: 2004 to 2025
- WOS.SSCI: 2015 to 2025

Searches:

1: ((TS=(Cerebral Palsy)) OR TS=(CP (Cerebral Palsy))) OR TS=(Little Disease)) OR TS=(Diplegia, Spastic)) OR TS=(Little's Disease)) OR TS=(Spastic Diplegia)) OR TS=(Diplegias, Spastic)) OR TS=(Spastic Diplegias)) OR TS=(Cerebral Palsy, Atonic)) OR TS=(Atonic Cerebral Palsy)) OR TS=(Cerebral Palsy, Hypotonic)) OR TS=(Hypotonic Cerebral Palsies)) OR TS=(Hypotonic Cerebral Palsy)) OR TS=(Cerebral Palsy, Athetoid)) OR TS=(Athetoid Cerebral Palsy)) OR TS=(Cerebral Palsies, Athetoid)) OR TS=(Cerebral Palsy, Dyskinetic)) OR TS=(Cerebral Palsies, Dyskinetic)) OR TS=(Dyskinetic Cerebral Palsy)) OR TS=(Monoplegic Cerebral Palsy)) OR TS=(Cerebral Palsies, Monoplegic)) OR TS=(Cerebral Palsy, Monoplegic)) OR TS=(Monoplegic Cerebral Palsies)) OR TS=(Cerebral

Palsy, Quadriplegic, Infantile)) OR TS=(Infantile Cerebral Palsy, Quadriplegic)) OR TS=(Quadriplegic Infantile Cerebral Palsy)) OR TS=(Cerebral Palsy, Congenital)) OR TS=(Congenital Cerebral Palsy)) OR TS=(Cerebral Palsy, Diplegic, Infantile)) OR TS=(Diplegic Infantile Cerebral Palsy)) OR TS=(Infantile Cerebral Palsy, Diplegic)) OR TS=(Cerebral Palsy, Dystonic-Rigid)) OR TS=(Cerebral Palsies, Dystonic-Rigid)) OR TS=(Cerebral Palsy, Dystonic Rigid)) OR TS=(Dystonic-Rigid Cerebral Palsies)) OR TS=(Dystonic-Rigid Cerebral Palsy)) OR TS=(Cerebral Palsy, Mixed)) OR TS=(Mixed Cerebral Palsies)) OR TS=(Mixed Cerebral Palsy)) OR TS=(Cerebral Palsy, Rolandic Type)) OR TS=(Rolandic Type Cerebral Palsy)) OR TS=(Cerebral Palsy, Spastic)) OR TS=(Spastic Cerebral Palsies)) OR TS=(Spastic Cerebral Palsy)) OR TS=(Cerebral Palsy, Monoplegic, Infantile)) OR TS=(Infantile Cerebral Palsy, Monoplegic)) OR TS=(Monoplegic Infantile Cerebral Palsy) Date Run: Mon Apr 07 2025 21:56:48 GMT+0800 (China Standard Time) Results: 541684

2: #[UNKNOWN SET REFERENCE] AND #1 Date Run: Mon Apr 07 2025 21:57:25 GMT+0800 (China Standard Time) Results: 2224

3: #[UNKNOWN SET REFERENCE] AND #1 and Clinical Neurology (Web of Science Categories) Date Run: Mon Apr 07 2025 21:57:52 GMT+0800 (China Standard Time) Results: 67

S3

Embase: 31

Session Results

....

No.	Query Results	Results	Date
#5.	#3 AND #4	31	7 Apr 2025
#4.	embedding	42,499	7 Apr 2025
#3.	#1 OR #2	58,289	7 Apr 2025
#2.	'brain palsy'/exp OR 'brain palsy' OR 'brain paralysis'/exp OR 'brain paralysis' OR 'central palsy'/exp OR 'central palsy' OR 'central paralysis'/exp OR 'central paralysis' OR 'cerebral paralysis'/exp OR 'cerebral paralysis' OR 'cerebral paresis'/exp OR 'cerebral paresis' OR 'diplegia spastica'/exp OR 'diplegia spastica' OR 'encephalopathia infantilis'/exp OR 'encephalopathia infantilis' OR 'palsy, cerebral'/exp OR 'palsy, cerebral' OR 'spastic diplegia'/exp OR 'spastic diplegia' OR 'cerebral palsy'/exp OR 'cerebral palsy'	58,289	7 Apr 2025
#1.	'cerebral palsy'/exp	51,745	7 Apr 2025

....

S4

Cochrane Library: 32

ID Search Hits

#1 MeSH descriptor: [Cerebral Palsy] explode all trees 2395

#2 Spastic Cerebral Palsies OR Cerebral Palsy, Spastic OR Spastic Cerebral Palsy OR Dystonic-Rigid Cerebral Palsies OR Cerebral Palsy, Dystonic Rigid OR Cerebral Palsies, Dystonic-Rigid OR Dystonic-Rigid Cerebral Palsy OR Cerebral Palsy, Dystonic-Rigid OR Rolandic Type Cerebral Palsy OR Cerebral Palsy, Rolandic Type OR Quadriplegic Infantile Cerebral Palsy OR Infantile Cerebral Palsy, Quadriplegic OR Cerebral Palsy, Quadriplegic, Infantile OR Athetoid Cerebral Palsy OR Cerebral Palsy, Athetoid OR Cerebral Palsies, Athetoid OR Dyskinetic Cerebral Palsy OR Cerebral Palsies, Dyskinetic OR Cerebral Palsy, Dyskinetic OR Cerebral Palsy, Mixed OR Mixed Cerebral Palsy OR Mixed Cerebral Palsies OR CP (Cerebral Palsy) OR Cerebral Palsy, Congenital OR Congenital Cerebral Palsy OR Monoplegic Cerebral Palsy OR Cerebral Palsy, Monoplegic OR Monoplegic Cerebral Palsies OR Cerebral Palsies, Monoplegic OR Cerebral Palsy, Diplegic, Infantile OR Infantile Cerebral Palsy, Diplegic OR Diplegic Infantile Cerebral Palsy OR Monoplegic Infantile Cerebral Palsy OR Cerebral Palsy, Monoplegic, Infantile OR Infantile Cerebral Palsy, Monoplegic OR Little Disease OR Diplegia, Spastic OR Diplegias, Spastic OR Little's Disease OR Spastic Diplegia OR Spastic Diplegias OR Hypotonic Cerebral Palsies OR Atonic Cerebral Palsy OR Hypotonic Cerebral Palsy OR Cerebral Palsy, Atonic OR Cerebral Palsy, Hypotonic 18267

#3 #1 OR #2 19088

#4 embedding 1271

#5 #3 AND #4 32
